# Locked-In Syndrome from Delayed Vertebral Artery Dissection After Strangulation: A Case Report

**DOI:** 10.5811/cpcem.63928

**Published:** 2026-09-20

**Authors:** Jennifer R. Snippen, Kien Huynh

**Affiliations:** * Lane Community College, Science Department, Eugene, Oregon; † Good Samaritan Regional Medical Center, Department of Internal Medicine, Corvallis, Oregon

**Keywords:** vertebral artery dissection, locked-in syndrome, strangulation, medicolegal, case report

## Abstract

**Introduction:**

Vertebral artery dissection is a rare but life-threatening complication of strangulation. Symptoms are often nonspecific and may not manifest immediately, complicating diagnosis. If prior neck trauma is not elicited during assessment, clinicians may miss a mechanistic link to prompt vascular imaging, leaving patients at risk for irreversible outcomes, as illustrated in this case.

**Case Report:**

A 24-year-old woman presented to a critical access emergency department (ED) with nonspecific neurological symptoms that had largely resolved upon arrival. Her only reported history was vague head and neck pain, which she attributed to stress after leaving an abusive relationship several weeks earlier. Hours later, while still in the ED, the patient experienced abrupt neurological deterioration. She was transferred to a regional medical center, where magnetic resonance imaging eventually revealed vertebral artery dissection with basilar artery hypoperfusion and extensive pontine infarction. Collateral history obtained from family revealed a preceding strangulation event. Police at the scene had documented neck injury, but the patient declined their offer of a medical assessment. By the time vertebral artery dissection was diagnosed, she had developed irreversible neurological damage leading to locked-in syndrome. She survived in this state for years before succumbing to complications of quadriplegia. The death was certified as a homicide due to inflicted blunt force neck injury.

**Conclusion:**

This case underscores the importance of timely, skilled medical assessment after strangulation and highlights the need for targeted screening of patients with vague neurological symptoms or disclosure of intimate partner violence.

## INTRODUCTION

Early recognition of vertebral artery dissection offers the greatest opportunity to minimize ischemic injury and salvage the viable penumbra.^1^ However, diagnosis can be challenging when symptoms are subtle, delayed, or mistaken for more benign conditions.^2^ Patients may not readily associate precipitating events with their symptoms and consequently may not disclose the events to clinicians.^3^ This case illustrates how failure to elicit disclosure of strangulation and obtain prompt vascular imaging may allow progression from subtle prodromal symptoms to irreversible, catastrophic injury. It highlights the importance of direct screening for strangulation and maintaining a high index of suspicion for vascular injury in patients presenting with vague or evolving neurologic symptoms.

## CASE REPORT

A 24-year-old woman with no significant past medical history, presented to a critical access emergency department (ED) by ambulance. To paramedics, she reported waking from a nap with headache, two bouts of emesis, and transient difficulty moving her left side. She also mentioned transient slurred speech and nonspecific visual difficulties.

Upon arrival in the ED, the original symptoms had largely resolved. The patient denied recent trauma and clinicians explicitly excluded motor vehicle collision, blunt head injury, or fall. She also denied difficulty breathing, swallowing, confusion or any problems with hearing or balance. The patient’s sole recent complaint was a history of persistent left-sided neck and shoulder pain, associated with a headache that seemed to worsen when she turned her head. She attributed these symptoms to the stress of leaving an abusive relationship weeks prior and expressed frustration, stating, “How many times do I have to tell this story; it’s so hard to talk.” Ice, massage, and over-the-counter medications had been used without much relief.

The patient was afebrile with a blood pressure of 133/88 mm Hg. All other vital signs were within normal limits. Physical examination revealed only tenderness in the suboccipital triangle and left levator scapula. Neurological exam was entirely normal with intact cranial nerves, full strength, normal tone, and symmetric deep tendon reflexes. Plantar stimulation elicited a normal flexion response. The neck was supple with no bony tenderness. Routine bloodwork was unremarkable.

The patient was sent for plain cervical films and non-contrast head computed tomography (CT), both of which were negative. Upon return from imaging, she abruptly developed seizure-like activity with decorticate posturing in the left arm and left gaze deviation with nystagmus. She became fully noncommunicative but responded to painful stimuli and periodically seemed to follow commands. Pupillary and gag reflexes remained intact. The patient was stabilized and transferred to a higher level of care for further imaging and neurology consultation. At the receiving facility, clinicians initially focused on ruling out new-onset seizures, viral meningitis, or meningoencephalitis. Meanwhile, her neurological status continued to deteriorate, requiring intubation.

Approximately nine hours after initial emergency medical services (EMS) contact, magnetic resonance imaging was performed, followed by magnetic resonance angiography. Together, these studies demonstrated an acute-to-subacute infarction involving most of the pons, with extension into the medulla and cerebellum, in the setting of a vertebrobasilar dissection and reduced flow in the left vertebral and basilar arteries.

Although the patient remained unresponsive, her family provided a critical detail: She had been strangled several weeks earlier. This disclosure triggered coordination among clinicians and law enforcement. Details emerged of an event, 32 days prior, in which the patient called EMS after being strangled. She reported being forced against a wall by a former partner as he compressed her anterior neck using some combination of his hands and an aluminum bat pressed horizontally across her throat. She experienced neck pain but denied loss of consciousness. Responding officers documented erythema on the anterior neck and offered to summon paramedics; the patient declined, choosing instead to leave and be with family.

*CPC-EM Capsule*

What do we already know about this clinical entity?

*Nonspecific or delayed symptoms of vertebral artery dissection (VAD) may complicate diagnosis.*

What makes this presentation of disease reportable?

*First reported case of VAD progressing to locked-in syndrome in an adult strangulation victim.*

What is the major learning point?

*Disclosure of interpersonal violence should prompt strangulation screening, and nonspecific neurologic symptoms warrant a high index of suspicion for VAD.*

How might this improve emergency medicine practice?

*Expanded awareness of potential strangulation sequelae may improve medical evaluation and refusal counseling.*

The patient’s family confirmed that she had been complaining of persistent neck and head pain since the event. They noted that her symptoms had progressively worsened, and she had begun to appear pale and fatigued. Medical care was not sought until the abrupt development of new neurological symptoms. By the time the strangulation history was elicited, the patient had already suffered irreversible brainstem infarction. She was ultimately discharged with locked-in syndrome—cognitively intact but ventilator-dependent and quadriplegic, able to communicate only through vertical eye movements and blinking. With time, she regained limited movement of her lips and was able to mouth words with some effectiveness. The patient survived for several years in this condition.

At the time of her death, the case was referred for medicolegal death investigation. A medical examiner certified the cause of death as urosepsis resulting from complications of an indwelling urinary catheter, as a consequence of quadriplegia due to vertebral artery dissection and pontine infarction, consequent to inflicted blunt force neck injury. The manner of death was classified as homicide.

## DISCUSSION

This case illustrates a convergence of multiple levels of failure in the recognition of vertebral artery dissection following strangulation. The patient’s initial refusal of medical care, combined with the nonspecific and evolving nature of her symptoms, and delayed disclosure of the strangulation mechanism obscured the underlying vascular injury. Collectively, these factors delayed diagnosis, allowing progression of ischemic injury and culminating in locked-in syndrome. Strangulation events can produce a wide spectrum of physiological effects that may be localized or systemic, transient or delayed, and span a continuum from seemingly insignificant to immediately fatal.^4^,^5^ Among the most serious sequelae is arterial intimal disruption, with carotid artery injury recognized as a cause of acute or delayed infarction and strangulation-related mortality.^5^–^7^

Vertebral artery involvement has been underrepresented in the strangulation literature, possibly in part because of perceived skeletal shielding afforded by the cervical vertebrae.^5^ Forceful neck rotation, flexion, or hyperextension may damage vertebral arteries as they ascend through the transverse foramina or and enter the skull near their basilar confluence.^8^ Such injuries have historically been associated with chiropractic manipulation, sports trauma, martial arts, and, more recently, percussive massage devices.^1^,^3^,^9^ Our review of the literature identified only one prior case of vertebral artery dissection following strangulation, reported in an adolescent victim.^10^ To our knowledge, this is the first reported case of vertebral artery dissection in an adult victim of strangulation and the first to progress to locked-in syndrome.

Nonspecific symptoms associated with VAD may precede infarction by days to weeks. These may include headache, neck pain, nausea, visual symptoms, paresthesias, cerebellar signs (eg ataxia, nystagmus), and lateral medullary signs (eg, vertigo, dysphagia, dysarthria).^2^,^3^ Delayed or evolving symptoms may complicate diagnosis, particularly when a precipitating event is not disclosed.^1^,^3^ Clinicians should neither assume that patients will disclose intimate partner strangulation nor expect them to attribute delayed-onset symptoms to a strangulation event without prompting.^11^,^12^ We also note that this patient’s evolving neurologic symptoms may have impaired her ability to effectively communicate with clinicians.

For detection of cervical vascular injury, computed tomography angiography (CTA) and magnetic resonance angiography are endorsed—with CTA favored as the initial modality in strangulation cases.^5^,^13^ As this case demonstrates, these critical studies may not be considered when strangulation is not disclosed or when clinicians fail to consider the possibility of underlying vascular injury.

Stabilization of acute strangulation should prioritize airway and cerebral perfusion, followed by evaluation for possible cervical vascular injury.^7^,^14^ Patients who are asymptomatic may be discharged with detailed return precautions; however; soft-tissue injury or neurologic symptoms may warrant observation and advanced screening.^14^ If vertebral artery dissection is diagnosed, the prognosis may be favorable.^1^,^3^,^9^,^15^ Although treatment approaches lack definitive consensus, current guidelines endorse antiplatelet or anticoagulant therapy, with endovascular or surgical intervention available as increasingly invasive options.^1^,^10^,^15^

These findings underscore the need for careful strangulation assessment in the context of reported interpersonal violence, coupled with a high index of suspicion for vertebral artery dissection in patients with nonspecific neurological symptoms. Disclosure of strangulation improves with direct screening, yet research indicates that frontline clinicians lack adequate training and require strangulation-specific protocols to improve recognition and response.^11^,^12^ Strangulation survivors who decline medical assessment should receive refusal counseling that includes an explicit warning about the possibility of delayed stroke. Law enforcement officials, who are often the first point of contact, should receive mandated, standardized training on the full scope of strangulation risks.^11^ The circumstances presented in this case indicate that family and community education may also contribute to early recognition and timely referral.

Finally, this case highlights the value of interdisciplinary collaboration among family members, clinicians, law enforcement, and medicolegal death investigators. Although delayed, the eventual coordination enabled accurate mechanism recognition, informed criminal justice proceedings, and ensured appropriate death certification. Standardized protocols and shared training across disciplines may improve patient outcomes, medicolegal accuracy, survivor safety, and strangulation surveillance.

This case report is limited by the inherent constraints of a retrospective single-case observation, including the completeness and availability of medical record data. Because of the time interval between the patient’s initial evaluation and submission of this case report, the original digital imaging archives were no longer available. Therefore, all imaging-based conclusions are derived from the written reports.

## CONCLUSION

This case illustrates several challenges in the recognition and management of strangulation-related vertebral artery dissection. The findings support routine strangulation screening in patients disclosing interpersonal violence, recognizing strangulation as a high-risk mechanism, and maintaining vigilance for cervical artery injury in patients presenting with nonspecific neurological symptoms.
